# Are We Germ Crazy?

**Published:** 1917-01

**Authors:** 


### Are We Germ Crazy?

About the time that the American people begin to think that they are making some progress in their fight on germs along comes some so-called scientist and tells them that the very things they have been doing all their lives to destroy germs has had the effect of increasing them. Years ago before the toothbrush was invented our forefathers used a green switch frayed at the end to clean the teeth with, something like the old-time snuff stick. They found that they swallowed too much of the splintered wood and that it frequently lodged and balled in the stomach and caused serious trouble. Then came the toothbrush, and through medical journals and in the newspapers, even in the schools—in fact, everywhere—people were told to use the toothbrush and use it regularly. Toothbrush drills were given, and fond mothers offered rewards to their children for the regular use of the toothbrush. It became a disgrace not to do so.

But, alas and alack! We are now being warned that the toothbrush is man's most deadly enemy, that it lays around all day seeking and devouring all sorts of germs with which to fill the mouth of its owner to his certain undoing. We are expected to believe that every time we use a toothbrush we fill our systems with germs. It seems there is nothing to be done but to sterilize the toothbrush every time it is used, if one would guard against germ destruction. But who is going to take the time to do it?

The public drinking cup was abolished because it was decided that it was uncleanly and a germ carrier, and the private drinking cup became popular. But it is a bit inconvenient for a person to have a drinking cup about him at all times. There arose advocates of the individual cup and saucer, knives and forks, plates and tableware of all kinds, people who would have had us carry our individual eating equipment along whenever invited out to dinner. One editor even urged the use of individual door knobs to avoid catching the millions of germs certain to be left by the hundreds who daily enter every door. Finally a happy idea occurred to some shrewd individual that gave promise of dispensing with the individual drinking cup, which couldn't be sterilized every time used. The result was the bubble fountain.

But, alas and alack again! We are told now that the bubble fountain has become the abiding place of streptococci germs and bacillus prodigiosus in great numbers. They say that the organisms get into the water stream of the fountain and dance there like a ball for from two to one hundred and thirty-five minutes. If the fountain is of the kind that is operated by hand and made to bubble at pleasure, it is worse still. Then the germs drop back on the metal part of the fountain and remain until the next victim comes along and turns on the water. It is recommended that the fountains be made to bubble at an angle so that the germs cannot play on top of the water or fall back, but should that be done the average person will put the whole fountain end of metal in his mouth and coat it with germs, tobacco, bad liquor and other objectionable things.

What is a fellow to do about it? He must try to live.

